# The Most Common Vitamin D Receptor Polymorphisms (*ApaI,* *FokI*, *TaqI*, *BsmI*, and *BglI*) in Children with Dental Caries: A Systematic Review and Meta-Analysis

**DOI:** 10.3390/children8040302

**Published:** 2021-04-15

**Authors:** Masoud Sadeghi, Amin Golshah, Mostafa Godiny, Roohollah Sharifi, Atefeh Khavid, Nafiseh Nikkerdar, Santosh Kumar Tadakamadla

**Affiliations:** 1 Medical Biology Research Center, Kermanshah University of Medical Sciences, Kermanshah 6714415185, Iran; sadeghi_mbrc@yahoo.com; 2 Students Research Committee, Kermanshah University of Medical Sciences, Kermanshah 6715847141, Iran; 3 Department of Orthodontics, School of Dentistry, Kermanshah University of Medical Sciences, Kermanshah 6713954658, Iran; amin.golshah@gmail.com; 4 Department of Endodontics, School of Dentistry, Kermanshah University of Medical Sciences, Kermanshah 6713954658, Iran; mostafa_goodin@yahoo.com (M.G.); roholahsharifi@gmail.com (R.S.); 5 Department of Oral and Maxillofacial Radiology, School of Dentistry, Kermanshah University of Medical Sciences, Kermanshah 6713954658, Iran; atefehkhavid@gmail.com (A.K.); n.nikkerdar@kums.ac.ir (N.N.); 6 School of Medicine and Dentistry & Menzies Health Institute Queensland, Griffith University, Gold Coast 4214, Australia

**Keywords:** dental caries, tooth decay, polymorphism, vitamin D, meta-analysis

## Abstract

Vitamin D participates in the calcification of enamel and dentin and the appropriate immune responses to oral microbial infections. We aimed to assess the association between the most common vitamin D receptor (*VDR*) polymorphisms (*ApaI,* *FokI*, *TaqI*, *BsmI*, and *BglI*) and the risk of dental caries in children. Methods: PubMed/MEDLINE, Cochrane Library, Web of Science, and Scopus databases were comprehensively searched until 19 January 2021. Meta-analysis with odds ratios as the effect estimate along with 95% confidence intervals and subgroup analysis were conducted using Review Manager 5.3 software. Publication bias and sensitivity analyses were conducted by Comprehensive Meta-Analysis, version 2.0 software. Results: Seventy-eight studies were retrieved from the databases, with nine studies included in the final analysis. Based on five genetic models, there was no association between *ApaI (rs7975232)*, *TaqI (rs731236)*, *BsmI (rs1544410)*, *FokI (rs2228570)*, and *BglI (rs739837)* polymorphisms and susceptibility to dental caries, except for the *FokI (rs10735810)* polymorphism. Conclusion: Among the *VDR* polymorphisms considered, an association was found between the *FokI (rs10735810)* polymorphism and the risk of dental caries, with a protective role of the f allele and ff genotype.

## 1. Introduction

Dental caries is considered a complex and multifactorial disease as well as one of the most common diseases in industrialized and developing countries [1]. In the world, early childhood caries is considered to be the most common oral health problem in children [2] and is the most common childhood disease [3]. The age-standardized prevalence of dental caries in deciduous and permanent teeth was 7.8% and 29.4% and the number of prevalent cases was 532 and 2302 million in 2017, respectively [4]. In most developed countries, the prevalence of dental caries is declining sharply, while in developing countries, it is increasing [1]. Several genes such as genes included in enamel development, immune response, and saliva function can be associated with susceptibility to caries [5]. A genome-wide meta-analysis [6] showed that consideration of the environment and aggregate genetic effects is more significant than specific genetic variants. A genome-wide association scan [7] reported that several genomic regions showed suggestive evidence for association with dental caries. The heritability of dental caries varies between 40 and 60% [8,9,10]. Vitamin D is a fat-soluble steroid that is essential for maintaining the body’s mineral balance [11], and it plays an important role in the calcification of enamel and dentin and the immune response to microbial infections of the mouth [12,13,14,15]. The function and biological activity of vitamin D are modulated by its interaction with the vitamin D receptor (*VDR*) protein [16], and the activity of the *VDR* protein is affected by polymorphisms of the *VDR* gene [17]. More than 200 polymorphisms of the *VDR* gene have been reported [18,19]. The *VDR* gene was found to impact the activity of a major metabolite of vitamin D, which participates in the formation of tooth enamel [18,20], which demonstrates its potential implication for dental caries risk [21,22,23]. The most common functional *VDR* polymorphisms found to be potentially involved in oral and systemic conditions are *BsmI*, *FokI*, *TaqI*, *BglI*, and *ApaI* [24]. *BsmI*, *TaqI*, and *ApaI* polymorphisms were found to influence *VDR* protein structure, with *FokI* also influencing the transcriptional activity translation [25]. The aim of this meta-analysis is to evaluate the association between these *VDR* polymorphisms (*ApaI, FokI*, *TaqI*, *BsmI*, and *BglI*) and susceptibility to dental caries in children.

## 2. Materials and Methods

This systematic review was performed according to the Preferred Reporting Items for Systematic Reviews and Meta-Analyses (PRISMA) protocols [26].

### 2.1. Data Sources and Literature Search

Searches in PubMed/MEDLINE, Cochrane Library, Web of Science, and Scopus databases were comprehensively performed until January 19, 2021, without any restrictions. The search strategies for each database are shown in Table 1 The titles and abstracts were checked by two authors (M.S. and S.K.T.) and any disagreement was resolved by consensus with a third author (A.G.). We also checked the references of all included studies to ensure no study was missed. 

### 2.2. Eligibility Criteria and Study Selection

The inclusion criteria were: (1) case–control studies focusing on the association between *VDR* polymorphisms and the risk of dental caries; (2) studies reporting *VDR* polymorphisms (*ApaI (rs7975232), FokI (rs10735810)*, *TaqI (rs731236)*, *BsmI (rs1544410)*, *FokI (rs2228570)*, and *BglI (rs739837)*) in children (age < 18 years); (3) dental caries confirmed by clinical examinations; (4) studies reporting the frequencies of alleles or genotypes; and (5) a control group with no tooth decay. Reviews, conference papers, and studies with no control group or those among adults or reporting other polymorphisms of *VDR* were excluded. The data from published studies were retrieved independently by two authors (M.S. and R.S.) to retrieve the necessary information. In case of discrepancies between the data extracted by the two authors, a duplicate data extraction was performed by a third author (M.G.).

### 2.3. Quality Assessment

Three reviewers (M.S., A.K., and N.N.) independently assessed the quality of the selected studies by scoring them according to Table 2. We developed a quality assessment tool specifically for this study, which consisted of 7 criteria. The range of scores varies from 0 to 11, with higher scores indicating better study quality.

### 2.4. Statistical Analysis

The association between polymorphisms and dental caries susceptibility was calculated by odds ratios (ORs) with 95% confidence intervals (CIs) based on five genetic models (allele, homozygote, heterozygote, recessive, and dominant models). To calculate heterogeneity, a chi-square-based Q test and the I^2^ statistic were used [27,28]. A *p*-value of > 0.10 and I^2^ < 50% indicated that there was no heterogeneity between the studies. However, considering the diversity in the effect sizes and populations between the studies, we used a random effects model in all analyses. Subgroup analysis (based on ethnicity and genotyping method) and sensitivity analysis (“one study removed” and “cumulative analysis”) were applied to find the effect of subgroups on the overall results and the stability of results, respectively. Funnel plots were used to determine publication bias. The *p*-value of (two-sided) < 0.05 was considered significant, but the size of the effect was also taken into consideration to determine the association between the polymorphism and dental caries. The forest plots and subgroup analysis were conducted by Review Manager 5.3 (RevMan 5.3) software, while publication bias and sensitivity analyses were performed using Comprehensive Meta-Analysis version 2.0 (CMA 2.0) software. The polymorphisms (*ApaI (rs7975232), FokI (rs10735810)*, *TaqI (rs731236)*, *BsmI (rs1544410)*, *FokI (rs2228570)*, and *BglI (rs739837)*) were demonstrated to not be in strong linkage disequilibrium (LD) with each other (r^2^ < 1) using the *LDlink* online tool *(*https://ldlink.nci.nih.gov) (accessed on 6 November 2020) [29], and therefore all polymorphisms were included in the present meta-analysis.

## 3. Results

### 3.1. Study Selection

**Figure 1:**
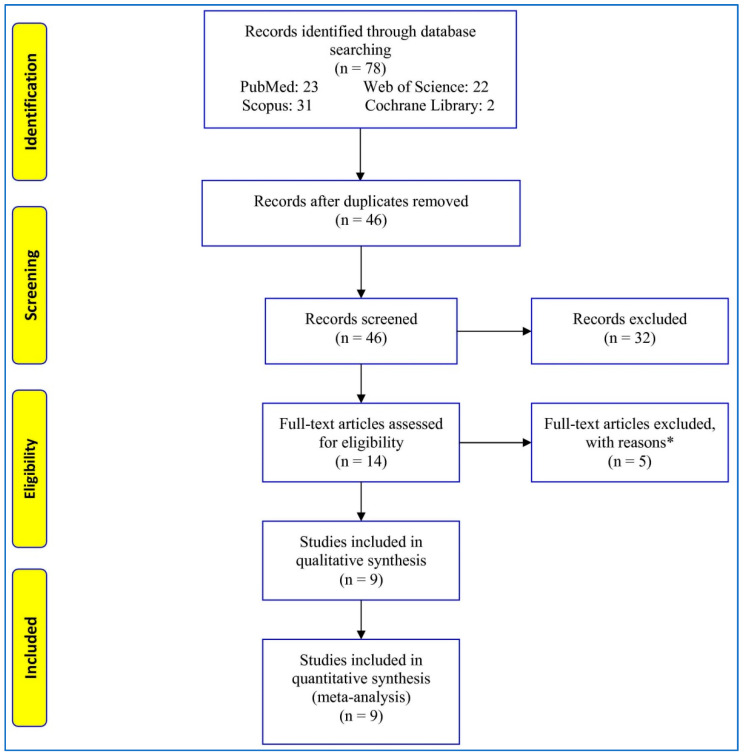
Flowchart of the study selection. * One article was a systematic review. One article had no control group. One article reported other vitamin D receptor (*VDR*) polymorphisms. Two articles reported *VDR* polymorphisms in adults.

Seventy-eight studies were retrieved from the databases (Figure 1). After removing and excluding duplicate and irrelevant records, 14 full texts were evaluated for eligibility. Then, five full-text articles were excluded for different reasons: one article was a systematic review, one article had no control group, one article reported other *VDR* polymorphisms, and two articles reported *VDR* polymorphisms in adults. At last, nine studies were included in the qualitative and quantitative analysis.

### 3.2. Quality Assessment

The seven criteria used for quality assessment are shown in Table 2. The maximum possible score was 11, while the minimum was 0.

### 3.3. Characteristics of Studies

Table 3 shows the characteristics of nine studies included in the meta-analysis [21,30,31,32,33,34,35,36,37]. Out of nine studies, three each were reported from China [21,36,37] and Brazil [31,33,35], and one each from Turkey [32], Czech Republic [34], and India [30]. There were three studies each on Caucasian, Asian, and mixed ethnic participants. The source of the control was population-based/school-based in all studies.

The prevalence of alleles and genotypes of six polymorphisms is shown in Table 4. In addition, the *p*-value of the Hardy–Weinberg equilibrium (HWE) for controls is reported.

### 3.4. Meta-Analysis

Table 5 shows the pooled analysis of the association between the *ApaI (rs7975232)* polymorphism and the risk of dental caries. The pooled ORs for allele, homozygote, heterozygote, recessive, and dominant were 0.89 (95%CI: 0.70, 1.13; *p* = 0.34; I^2^ = 52%], 0.86 (95%CI: 0.49, 1.50; *p* = 0.59; I^2^ = 57%], 0.83 (95%CI: 0.42, 1.62; *p* = 0.58; I^2^ = 69%], 0.91 (95%CI: 0.55, 1.50; *p* = 0.71; I^2^ = 50%], and 0.87 (95%CI: 0.69, 1.10; *p* = 0.24; I^2^ = 8%], respectively. These results indicate that there was no association between the *ApaI (rs7975232)* polymorphism and susceptibility to dental caries.

Table 6 demonstrates that the f allele (0.58 (95%CI: 0.38, 0.88); *p* = 0.01; I^2^ = 85%), homozygote (0.52 (95%CI: 0.29, 0.92; *p* = 0.02; I^2^ = 66%), and dominant models (0.53 (95%CI: 0.33, 0.87; *p* = 0.01; I^2^ = 64%) of the *FokI (rs10735810)* ff genotype polymorphism had a protective role for the risk of dental caries, and the likelihood of caries in the individuals with these polymorphisms was approximately half that of those without these polymorphisms. The pooled ORs for other genetic models of *FokI (rs10735810)* polymorphisms (heterozygote and recessive) were not significant and the effect estimate was nearer to 1.

There was no association between the *TaqI (rs731236)* polymorphism and susceptibility to dental caries based on the five genetic models (Table 7).

Table 8 shows that the pooled ORs for allele, homozygote, heterozygote, recessive, and dominant were 0.92 (95%CI: 0.58, 1.46; *p* = 0.73; I^2^ = 68%], 3.89 (95%CI: 0.16, 95.85; *p* = 0.41], 2.71 (95%CI: 0.11, 69.34; *p* = 0.55], 3.74 (95%CI: 0.15, 92.12; *p* = 0.42], and 0.86 (95%CI: 0.48, 1.54; *p* = 0.61; I^2^ = 76%], respectively. Although the effect estimates for homozygote, heterozygote, and recessive models were >1, these estimates were derived from only one study each, and wider confidence intervals indicate that the sample sizes in these studies were very small. These findings indicate that there was no association between the *BsmI (rs1544410)* polymorphism and susceptibility to dental caries.

Table 9 demonstrates that there was no association between the *FokI (rs2228570)* polymorphism and susceptibility to dental caries and there was a lack of heterogeneity between the studies (I^2^ = 0%) in all five genetic models. The odds ratio for most of these models was closer to 1, with narrow confidence intervals indicating no association.

The pooled ORs for allele, homozygote, heterozygote, recessive, and dominant were 1.06 (95%CI: 0.86, 1.31; *p* = 0.61; I^2^ = 0%], 1.15 (95%CI: 0.75, 1.75; *p* = 0.53; I^2^ = 0%], 1.15 (95%CI: 0.79, 1.67; *p* = 0.48; I^2^ = 0%], 1.14 (95%CI: 0.80, 1.62; *p* = 0.46; I^2^ = 0%], and 1.02 (95%CI: 0.73, 1.42; *p* = 0.91; I^2^ = 0%], respectively (Table 10). There was no association between the *BglI (rs739837)* polymorphism and susceptibility to dental caries.

### 3.5. Subgroup Analysis

As there was an adequate number of studies on the *TaqI (rs731236)* polymorphism, subgroup analyses in relation to ethnicity and genotyping were conducted (Table 11). The overall effect still remained insignificant with none of the subgroups demonstrating any association between the *TaqI (rs731236)* polymorphism and susceptibility to dental caries across the five genetic models.

### 3.6. Sensitivity Analysis

We conducted “cumulative analysis” and “one study removed” analyses to evaluate the stability of the findings related to six polymorphisms. The results show that the results were consistent/stable for the six polymorphisms. Additionally, for the *TaqI (rs731236)* polymorphism, we removed two studies [34,36] reporting an HWE deviation in the control group and found that the pooled ORs still remained the same.

### 3.7. Publication Bias

**Figure 2:**
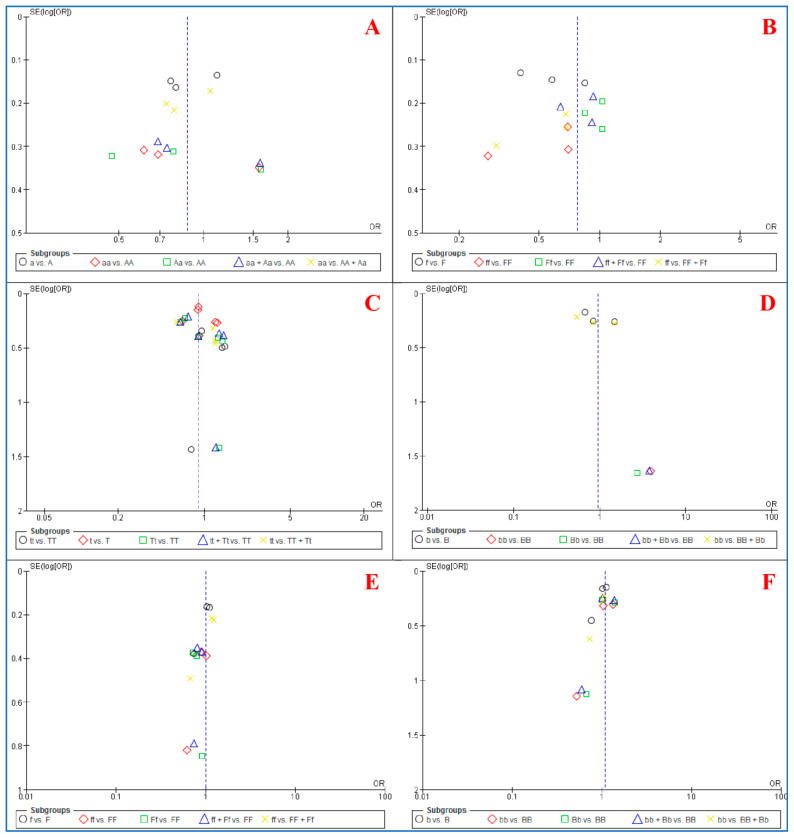
Funnel plots for association between six polymorphisms of vitamin D receptor (*VDR*) and dental caries risk based on five genetic. (**A**): *ApaI (rs7975232)*. (**B**): *FokI (rs10735810)*. (**C**): *TaqI (rs731236)*. (**D**): *BsmI (rs1544410)*. (**E**): *FokI (rs2228570)*. (**F**): *BglI (rs739837)*.

The funnel plots (Figure 2) and *p* > 0.05 for both Egger’s and Begg’s tests demonstrate a lack of publication bias with regard to all six polymorphisms considered in this review.

## 4. Discussion

The present meta-analysis evaluated the association between *VDR* polymorphisms (*ApaI (rs7975232), FokI (rs10735810)*, *TaqI (rs731236)*, *BsmI (rs1544410)*, *FokI (rs2228570)*, and *BglI (rs739837)*) and the risk of dental caries in children. None of the polymorphisms were associated with the risk of dental caries, except for the *FokI (rs10735810)* polymorphism, with the f allele and ff genotype of this polymorphism having a protective role in dental caries occurrence.

The role of genetic factors in the risk of dental caries is still largely unknown despite numerous studies. Dental caries is a multifactorial disease caused by interactions between environmental factors, behavioral factors, several genetic factors, and gene–environment interactions [31]. Advances in transcriptional research have provided a variety of data on the interaction between *VDR* and other transcriptionally active proteins, demonstrating the potential of *VDR* to exert a wide range of biological reactions [38]. Vitamin D is known as a modulator of calcium homeostasis and plays an important role in regulating electrolytes and blood pressure. Evidence has shown that the most active metabolite of this vitamin can regulate the immune response and also has anti-inflammatory activity [39]. *VDR* gene polymorphisms have been shown to be strongly related to mineral density [32,40,41] and a meta-analysis [42] confirmed this. Although results from individual studies remain inconsistent, a meta-analysis of controlled clinical trials showed that early vitamin D supplementation could reduce the risk of dental caries by 47–54% [20]. Although the mechanism of action is unknown, *VDR* gene polymorphisms could modulate the effect of vitamin D supplementation. For instance, one study found some *VDR* polymorphisms to modify the association of vitamin D supplementation with the risk of a specific type of cancer [43]. The role of *VDR* polymorphisms in modifying the effect of vitamin D supplementation on dental caries needs further exploration.

*VDR* plays an important role in regulating the expression of genes associated with the immune response, calcium homeostasis, and cell differentiation and proliferation [18]. The distribution of *VDR* polymorphisms could show different patterns based on ethnicities and age [44,45,46,47]. Research has shown ethnic differences in vitamin D status and their correlation to hormonal homeostasis and bone phenotype, as well as the influence of environmental factors such as lifestyle, diet, and sun exposure [17,18]. However, we could not find any differences based on ethnicities in this meta-analysis.

**Figure 3:**
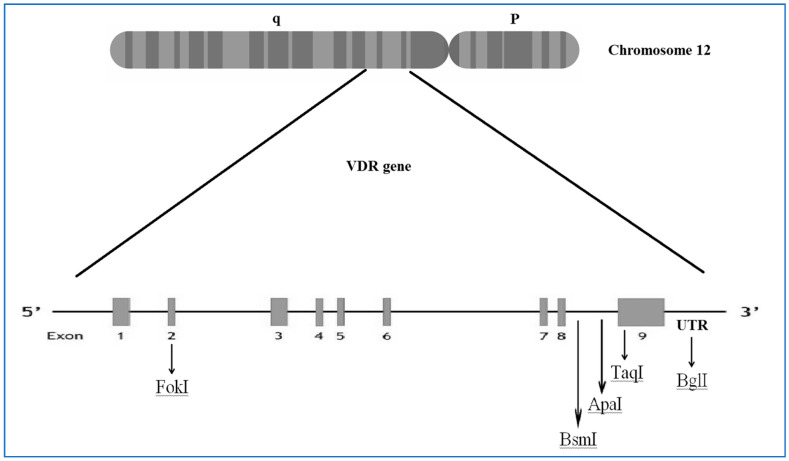
The location of vitamin D receptor (*VDR*) polymorphisms reported in the meta-analysis.

Our meta-analysis showed a protective role of the FokI (rs10735810) polymorphism on dental caries. This might be due to its interactions with co-transcription factors [18] and its location (Figure 3) [18,48].

The meta-analysis has several limitations and strengths. Limitations include the presence of fewer published reports on this topic hindering the performance of any meta-regression analysis, studies with small sample sizes, and clinical and statistical heterogeneity between the studies. Some studies included in the meta-analysis did not match cases with controls, used genotyping methods different from other studies, and had controls with a deviation of the HWE. It also needs mentioning that we could not conduct any analysis to adjust the effect of multiple testing or multiplicity within the included studies. Despite the limitations, this review demonstrates several strengths in the form of the lack of publication bias, the suitable quality of all the included studies, and the use a population-based source for recruiting controls in all the studies. More studies on larger sample sizes and different ethnicities will help to explore the influence of different *VDR* polymorphisms on the risk of dental caries.

## 5. Conclusions

Out of the six *VDR* polymorphisms explored in this meta-analysis, an association was only observed between the *FokI (rs10735810)* polymorphism and the risk of dental caries, with the f allele and ff genotype demonstrating a protective role in the occurrence of dental caries.

## Figures and Tables

**Table 1:** Search strategies.

Database	Search
PubMed	(“Vit D”[Title/Abstract] OR “Vitamin D” [Title/Abstract] OR “calciferol”[Title/Abstract] OR “VDR” [Title/Abstract]) AND (“dental caries” [Title/Abstract] OR “caries”[Title/Abstract] OR “decay”[Title/Abstract]) AND (“gene”[Title/Abstract] OR “polymorphism*”[Title/Abstract] OR “variant*”[Title/Abstract] OR “allele*”[Title/Abstract] OR “genetic*”[Title/Abstract])
Cochrane Library	(“Vit D”:ti,ab,kw OR “Vitamin D”:ti,ab,kw OR “calciferol”:ti,ab,kw OR “VDR”:ti,ab,kw) AND (“dental caries”:ti,ab,kw OR “caries”:ti,ab,kw OR “decay”:ti,ab,kw) AND (“polymorphism*”:ti,ab,kw OR “variant*”:ti,ab,kw OR “genotype*”)
Web of Science	TS = (“Vit D” OR “Vitamin D” OR “calciferol” OR “VDR”) AND TS = (“dental caries” OR “caries” OR “decay”) AND TS = (“ polymorphism*” OR “variant*” OR “allele*” OR “genotype*”)
Scopus	(TITLE-ABS-KEY (“Vit D”) OR TITLE-ABS-KEY (“Vitamin D”) OR TITLE-ABS-KEY (“calciferol”) OR TITLE-ABS-KEY (“VDR”)) AND (TITLE-ABS-KEY (“dental caries”) OR TITLE-ABS-KEY (“caries”) OR TITLE-ABS-KEY (“decay”)) AND (TITLE-ABS-KEY (“polymorphism*”) OR TITLE-ABS-KEY (“variant*”) OR TITLE-ABS-KEY (“allele*”) OR TITLE-ABS-KEY (“genotype*”))

**Table 2:** Criteria for quality assessment.

Criteria	Score
1. Representativeness of cases	
Consecutive/randomly selected from case population with clearly defined sampling frame	2
Consecutive/randomly selected from case population without clearly defined sampling frame or with extensive inclusion/exclusion criteria	1
Not described	0
2. Source of controls	
Population- or community-based	2
Hospital-based	1
Not described	0
3. Ascertainment of dental caries	
Clinical examination	2
Diagnosis of caries by patient medical record	1
Not described	0
4. Sample size	
>1000	2
200–1000	1
<200	0
5. Age and sex were matched between cases and controls	
Yes	1
No/Not described	0
6. Quality control of genotyping methods	
Repetition of partial/total tested samples	1
Not described	0
7. Hardy–Weinberg equilibrium in control subjects	
Hardy–Weinberg equilibrium	1
Hardy–Weinberg disequilibrium	0

**Table 3:** Background characteristics of studies included in the meta-analysis.

First Author, Publication Year	Country	Ethnicity	Source of Control	Genotyping Method	Quality Score
Cogulu, 2016 [32]	Turkey	Caucasian	Population-based	PCR-RFLP	7
Holla, 2017 [34]	Czech Republic	Caucasian	Population-based	TaqMan	9
Kong, 2017 [21]	China	Asian	School-based	PCR	8
Yu, 2017 [37]	China	Asian	School-based	PCR-RFLP	10
Qin, 2019 [36]	China	Asian	Population-based	TaqMan	10
Aribam, 2020 [30]	India	Caucasian	Population-based	PCR	9
Barbosa, 2020 [31]	Brazil	Mixed	School-based	Real-Time PCR	8
Fatturi, 2020 [33]	Brazil	Mixed	School-based	Real-Time PCR	10
Madalena, 2020 [35]	Brazil	Mixed	School-based	Real-Time PCR	9

Abbreviations: PCR, polymerase chain reaction; RFLP, restriction fragment length polymorphism.

**Table 4:** Prevalence of alleles and genotypes of the polymorphisms in cases and controls.

First Author, Publication Year	Groups (*N*)	*ApaI (rs7975232)*			*FokI (rs10735810)*			*TaqI (rs731236)*			*BsmI (rs1544410)*			*p*-Value of HWE
		AA	Aa	aa	FF	Ff	ff	TT	Tt	Tt	BB	Bb	bb	
Cogulu, 2016 [32]	Case (112)	-	-	-	-	-	-	35	46	31	-	-	-	0.132
	Control (38)	-	-	-	-	-	-	15	14	9	-	-	-	
Holla, 2017 [34]	Case (235)	-	-	-	-	-	-	95	110	30	-	-	-	0.037
	Control (153)	-	-	-	-	-	-	51	85	17	-	-	-	
Kong, 2017 [21]	Case (249)	44	87	118	69	132	48	230	19	0	0	152	97	0.011, 0.662, 0.615, and <0.001
	Control (131)	18	43	70	34	63	34	120	11	0	0	60	71	
Yu, 2017 [37]	Case (200)	33	85	82	86	96	18	171	29	0	0	36	164	0.210, 0.057, 0.097, and 0.399
	Control (200)	24	79	97	65	86	49	158	42	0	0	31	169	
Qin, 2019 [36]	Case (304)	17	129	158	98	160	46	1	274	29	0	28	276	0.895, 0.764, <0.001, and 0.909
	Control (245)	21	100	124	75	119	51	1	207	37	1	31	213	
Aribam, 2020 [30]	Case (60)	-	-	-	-	-	-	22	25	13	-	-	-	0.158
	Control (60)	-	-	-	-	-	-	26	23	11	-	-	-	
First Author, Publication Year	Groups (*N*)	*FokI (rs2228570)*						*BglI (rs739837)*						*p*-Value of HWE
		FF		Ff		Ff		BB		Bb		bb		
Barbosa, 2020 [31]	Case (164 and 163)	19		64		81		29		82		52		0.691 and 0.347
	Control (179 and 188)	17		80		82		43		87		58		
Fatturi, 2020 [33]	Case (204 and 213)	22		85		97		63		101		49		0.435 and 0.692
	Control (132 and 121)	13		63		56		36		58		27		
Madalena, 2020 [35]	Case (138 and 99)	19		60		59		13		52		34		0.649 and 0.665
	Control (19 and 12)	2		7		10		1		6		5		

Abbreviation: HWE, Hardy–Weinberg equilibrium. AA, FF, TT, BB—homozygous dominant; Aa, Ff, Tt, Bb—heterozygous; aa, ff, bb—homozygous recessive.

**Table 5:** The results of pooled analysis for association between *ApaI (rs7975232)* polymorphism and dental caries risk based on five genetic models.

Genetic Model	First Author, Publication Year	Case		Control		Weight	Odds Ratio
		Events	Total	Events	Total		M-H, Random, 95%CI
a vs. A	Kong, 2017 [21]	323	498	183	262	30.3%	0.80 [0.58, 1.10]
	Yu, 2017 [37]	249	400	273	400	33.4%	0.77 [0.57, 1.03]
	Qin, 2019 [36]	445	608	348	490	36.4%	1.11 [0.85, 1.45]
Subtotal (95%CI)			1506		1152	100.0%	0.89 [0.70, 1.13]
Total events		1017		804			
Heterogeneity: Tau² = 0.02; Chi² = 4.19, df = 2 (*P =* 0.12); I² = 52%; Test for overall effect: *Z* = 0.95 (*p* = 0.34)							
aa vs. AA	Kong, 2017 [21]	118	162	70	88	33.9%	0.69 [0.37, 1.29]
	Yu, 2017 [37]	82	115	97	121	34.8%	0.61 [0.34, 1.12]
	Qin, 2019 [36]	158	175	124	145	31.3%	1.57 [0.80, 3.11]
Subtotal (95%CI)			452		354	100.0%	0.86 [0.49, 1.50]
Total events		358		291			
Heterogeneity: Tau² = 0.14; Chi² = 4.68, df = 2 (*P =* 0.10); I² = 57%; Test for overall effect: *Z* = 0.54 (*p* = 0.59)							
Aa vs. AA	Kong, 2017 [21]	87	164	43	61	33.7%	0.47 [0.25, 0.89]
	Yu, 2017 [37]	85	118	79	103	34.4%	0.78 [0.43, 1.44]
	Qin, 2019 [36]	129	146	100	121	31.8%	1.59 [0.80, 3.18]
Subtotal (95% CI)			428		285	100.0%	0.83 [0.42, 1.62]
Total events		301		222			
Heterogeneity: Tau² = 0.24; Chi² = 6.50, df = 2 (*P =* 0.04); I² = 69%; Test for overall effect: *Z* = 0.55 (*p* = 0.58)							
aa + Aa vs. AA	Kong, 2017 [21]	205	249	113	131	34.0%	0.74 [0.41, 1.34]
	Yu, 2017 [37]	167	200	176	200	35.6%	0.69 [0.39, 1.22]
	Qin, 2019 [36]	287	304	224	245	30.4%	1.58 [0.82, 3.07]
Subtotal (95%CI)			753		576	100.0%	0.91 [0.55, 1.50]
Total events		659		513			
Heterogeneity: Tau² = 0.10; Chi² = 4.03, df = 2 (*P =* 0.13); I² = 50%; Test for overall effect: *Z* = 0.37 (*p* = 0.71)							
aa vs. AA + Aa	Kong, 2017 [21]	118	249	70	131	28.2%	0.78 [0.51, 1.20]
	Yu, 2017 [37]	82	200	97	200	33.4%	0.74 [0.50, 1.10]
	Qin, 2019 [36]	158	304	124	245	38.5%	1.06 [0.75, 1.48]
Subtotal (95%CI)			753		576	100.0%	0.87 [0.69, 1.10]
Total events		358		291			
Heterogeneity: Chi² = 2.16, df = 2 (*P =* 0.34); I² = 8%; Test for overall effect: *Z* = 1.18 (*p* = 0.24)							

Abbreviation: CI, confidence interval.

**Table 6:** Meta-analysis for association between *FokI (rs10735810)* polymorphism and dental caries risk based on five genetic models.

Genetic Model	First Author, Publication Year	Case		Control		Weight	Odds Ratio
		Events	Total	Events	Total		M-H, Random, 95%CI
f vs. F	Kong, 2017 [21]	228	498	131	262	32.7%	0.84 [0.63, 1.14]
	Yu, 2017 [37]	132	400	184	400	33.1%	0.58 [0.43, 0.77]
	Qin, 2019 [36]	152	608	221	490	34.2%	0.41 [0.31, 0.52]
Subtotal (95%CI)			1506		1152	100.0%	0.58 [0.38, 0.88]
Total events		512		536			
Heterogeneity: Tau² = 0.12; Chi² = 13.37, df = 2 (*P =* 0.001); I² = 85%; Test for overall effect: *Z* = 2.56 (*p* = 0.01)							
ff vs. FF	Kong, 2017 [21]	48	117	34	68	32.3%	0.70 [0.38, 1.27]
	Yu, 2017 [37]	18	104	49	114	31.3%	0.28 [0.15, 0.52]
	Qin, 2019 [36]	46	144	51	126	36.4%	0.69 [0.42, 1.14]
Subtotal (95%CI)			365		308	100.0%	0.52 [0.29, 0.92]
Total events		112		134			
Heterogeneity: Tau² = 0.17; Chi² = 5.91, df = 2 (*P =* 0.05); I² = 66%; Test for overall effect: *Z* = 2.24 (*p* = 0.02)							
Ff vs. FF	Kong, 2017 [21]	132	201	63	97	23.3%	1.03 [0.62, 1.72]
	Yu, 2017 [37]	96	182	86	151	35.5%	0.84 [0.55, 1.30]
	Qin, 2019 [36]	160	258	119	194	41.2%	1.03 [0.70, 1.51]
Subtotal (95% CI)			641		442	100.0%	0.96 [0.75, 1.24]
Total events		388		268			
Heterogeneity: Chi² = 0.54, df = 2 (*P =* 0.76); I² = 0; Test for overall effect: *Z* = 0.29 (*p* = 0.77)							
ff + Aa vs. FF	Kong, 2017 [21]	180	249	97	131	22.9%	0.91 [0.57, 1.48]
	Yu, 2017 [37]	114	200	135	200	37.7%	0.64 [0.42, 0.96]
	Qin, 2019 [36]	206	304	170	245	39.4%	0.93 [0.65, 1.33]
Subtotal (95%CI)			753		576	100.0%	0.82 [0.64, 1.04]
Total events		500		402			
Heterogeneity: Chi² = 2.09, df = 2 (*P =* 0.35); I² = 4%; Test for overall effect: *Z* = 1.66 (*p* = 0.10)							
ff vs. FF + Ff	Kong, 2017 [21]	48	249	34	131	33.6%	0.68 [0.41, 1.13]
	Yu, 2017 [37]	18	200	49	200	29.9%	0.30 [0.17, 0.55]
	Qin, 2019 [36]	46	304	51	245	36.6%	0.68 [0.44, 1.05]
Subtotal (95%CI)			753		576	100.0%	0.53 [0.33, 0.87]
Total events			753		576	100.0%	0.53 [0.33, 0.87]
Heterogeneity: Tau² = 0.12; Chi² = 5.53, df = 2 (*P =* 0.06); I² = 64%; Test for overall effect: *Z* = 2.53 (*p* = 0.01)							

Abbreviation: CI, confidence interval.

**Table 7:** Association between *TaqI (rs731236)* polymorphism and dental caries risk based on five genetic models.

Genetic Model	First Author, Publication Year	Case		Control		Weight	Odds Ratio
		Events	Total	Events	Total		M-H, Random, 95%CI
t vs. T	Cogulu, 2016 [32]	108	224	32	76	7.3%	1.28 [0.76, 2.16]
	Holla, 2017 [34]	170	470	119	306	27.3%	0.89 [0.66, 1.20]
	Kong, 2017 [21]	19	498	11	262	4.1%	0.91 [0.42, 1.93]
	Yu, 2017 [37]	29	400	42	400	11.6%	0.67 [0.41, 1.09]
	Qin, 2019 [36]	332	608	281	490	42.0%	0.89 [0.70, 1.14]
	Aribam, 2020 [30]	51	120	45	120	7.7%	1.23 [0.73, 2.07]
Subtotal (95%CI)			2320		1654	100.0%	0.92 [0.79, 1.08]
Total events		709		530			
Heterogeneity: Chi² = 4.47, df = 5 (*P =* 0.48); I² = 0%; Test for overall effect: *Z* = 1.03 (*p* = 0.30)							
tt vs. TT	Cogulu, 2016 [32]	31	66	9	24	22.2%	1.48 [0.57, 3.85]
	Holla, 2017 [34]	30	125	17	68	53.1%	0.95 [0.48, 1.88]
	Kong, 2017 [21]	0	230	0	120		Not estimable
	Yu, 2017 [37]	0	171	0	158		Not estimable
	Qin, 2019 [36]	29	30	37	38	3.4%	0.78 [0.05, 13.07]
	Aribam, 2020 [30]	13	35	11	37	21.3%	1.40 [0.52, 3.73]
Subtotal (95%CI)			657		445	100.0%	1.15 [0.72, 1.86]
Total events		103		74			
Heterogeneity: Chi² = 0.79, df = 3 (*P =* 0.85); I² = 0%; Test for overall effect: *Z* = 0.58 (*p* = 0.56)							
Tt vs. TT	Cogulu, 2016 [32]	46	81	14	29	7.6%	1.41 [0.60, 3.30]
	Holla, 2017 [34]	110	205	85	136	40.5%	0.69 [0.45, 1.08]
	Kong, 2017 [21]	19	249	11	131	11.4%	0.90 [0.42, 1.96]
	Yu, 2017 [37]	29	200	42	200	30.7%	0.64 [0.38, 1.07]
	Qin, 2019 [36]	274	275	207	208	0.7%	1.32 [0.08, 21.29]
	Aribam, 2020 [30]	25	47	23	49	9.0%	1.28 [0.58, 2.86]
Subtotal (95% CI)			1057		753	100.0%	0.81 [0.62, 1.07]
Total events		503		382			
Heterogeneity: Chi² = 4.36, df = 5 (*P =* 0.50); I² = 0%; Test for overall effect: *Z* = 1.49 (*p* = 0.14)							
tt + Tt vs. TT	Cogulu, 2016 [32]	77	112	23	38	8.7%	1.43 [0.67, 3.08]
	Holla, 2017 [34]	140	235	102	153	40.5%	0.74 [0.48, 1.13]
	Kong, 2017 [21]	19	249	11	131	10.8%	0.90 [0.42, 1.96]
	Yu, 2017 [37]	29	200	42	200	29.1%	0.64 [0.38, 1.07]
	Qin, 2019 [36]	303	304	244	245	0.7%	1.24 [0.08, 19.96]
	Aribam, 2020 [30]	38	60	34	60	10.1%	1.32 [0.63, 2.75]
Subtotal (95%CI)			1160		827	100.0%	0.85 [0.66, 1.11]
Total events		606		456			
Heterogeneity: Chi² = 4.89, df = 5 (*P =* 0.43); I² = 0%; Test for overall effect: *Z* = 1.21 *p* = 0.23)							
tt vs. TT + Tt	Cogulu, 2016 [32]	31	112	9	38	13.2%	1.23 [0.52, 2.90]
	Holla, 2017 [34]	30	235	17	153	24.5%	1.17 [0.62, 2.21]
	Kong, 2017 [21]	0	249	0	131		Not estimable
	Yu, 2017 [37]	0	200	0	200		Not estimable
	Qin, 2019 [36]	29	304	37	245	50.5%	0.59 [0.35, 1.00]
	Aribam, 2020 [30]	13	60	11	60	11.7%	1.23 [0.50, 3.02]
Subtotal (95%CI)			1160		827	100.0%	0.93 [0.62, 1.40]
Total events		103		74			
Heterogeneity: Chi² = 4.14, df = 3 (*P =* 0.25); I² = 28; Test for overall effect: *Z* = 0.72 (*p* = 0.35)							

Abbreviation: CI, confidence interval.

**Table 8:** The results of meta-analysis exploring the association between *BsmI (rs1544410)* polymorphism and dental caries risk based on five genetic models.

Genetic Model	First Author, Publication Year	Case		Control		Weight	Odds ratio
		Events	Total	Events	Total		M-H, Random, 95%CI
b vs. B	Kong, 2017 [21]	346	498	202	262	38.6%	0.68 [0.48, 0.96]
	Yu, 2017 [37]	364	400	369	400	31.1%	0.85 [0.51, 1.40]
	Qin, 2019 [36]	580	608	457	490	30.3%	1.50 [0.89, 2.51]
Subtotal (95%CI)			1506		1152	100.0%	0.92 [0.58, 1.46]
Total events		1290		1028			
Heterogeneity: Tau² = 0.11; Chi² = 6.24, df = 2 (*P =* 0.04); I² = 68%; Test for overall effect: *Z* = 0.34 (*p* = 0.73)							
bb vs. BB	Kong, 2017 [21]	97	97	71	71		Not estimable
	Yu, 2017 [37]	164	164	169	169		Not estimable
	Qin, 2019 [36]	276	276	213	214	100.0%	3.89 [0.16, 95.85]
Subtotal (95%CI)			537		454	100.0%	3.89 [0.16, 95.85]
Total events		537		453			
Heterogeneity: Not applicable; Test for overall effect: *Z* = 0.83 (*p* = 0.41)							
Bb vs. BB	Kong, 2017 [21]	152	152	60	60		Not estimable
	Yu, 2017 [37]	36	36	31	31		Not estimable
	Qin, 2019 [36]	28	28	31	32	100.0%	2.71 [0.11, 69.34]
Subtotal (95% CI)			216		123	100.0%	2.71 [0.11, 69.34]
Total events		216		122			
Heterogeneity: Not applicable; Test for overall effect: *Z* = 0.60 (*p* = 0.55)							
bb + Bb vs. BB	Kong, 2017 [21]	249	249	131	131		Not estimable
	Yu, 2017 [37]	200	200	200	200		Not estimable
	Qin, 2019 [36]	304	304	244	245	100.0%	3.74 [0.15, 92.12]
Subtotal (95%CI)			753		576	100.0%	3.74 [0.15, 92.12]
Total events		753		575			
Heterogeneity: Not applicable; Test for overall effect: *Z* = 0.81 (*p* = 0.42)							
bb vs. BB + Bb	Kong, 2017 [21]	97	249	71	131	35.6%	0.54 [0.35, 0.83]
	Yu, 2017 [37]	164	200	169	200	32.4%	0.84 [0.49, 1.41]
	Qin, 2019 [36]	276	304	213	245	32.0%	1.48 [0.86, 2.54]
Subtotal (95%CI)			753		576	100.0%	0.86 [0.48, 1.54]
Total events		537		453			
Heterogeneity: Tau² = 0.20; Chi² = 8.32, df = 2 (*P =* 0.02); I² = 76%; Test for overall effect: *Z* = 0.51 (*p* = 0.61)							

Abbreviation: CI, confidence interval.

**Table 9:** Results exploring the association between *FokI (rs2228570)* polymorphism and dental caries risk based on five genetic models.

Genetic Model	First Author, Publication Year	Case		Control		Weight	Odds Ratio
		Events	Total	Events	Total		M-H, Random, 95%CI
f vs. F	Barbosa, 2020 [31]	226	328	244	358	46.3%	1.04 [0.75, 1.43]
	Fatturi, 2020 [33]	279	408	175	264	42.9%	1.10 [0.79, 1.53]
	Madalena, 2020 [35]	178	276	27	38	10.8%	0.74 [0.35, 1.56]
Subtotal (95%CI)			1012		660	100.0%	1.03 [0.83, 1.28]
Total events		683		446			
Heterogeneity: Chi² = 0.91, df = 2 (*P =* 0.63); I² = 0%; Test for overall effect: *Z* = 0.29 (*p* = 0.77)							
ff vs. FF	Barbosa, 2020 [31]	81	100	82	99	47.5%	0.88 [0.43, 1.82]
	Fatturi, 2020 [33]	97	119	56	69	39.7%	1.02 [0.48, 2.19]
	Madalena, 2020 [35]	59	78	10	12	12.8%	0.62 [0.12, 3.09]
Subtotal (95%CI)			297		180	100.0%	0.91 [0.55, 1.50]
Total events		237		148			
Heterogeneity: Chi² = 0.32, df = 2 (*P =* 0.85); I² = 0%; Test for overall effect: *Z* = 0.37 (*p* = 0.71)							
Ff vs. FF	Barbosa, 2020 [31]	64	83	80	97	48.2%	0.72 [0.34, 1.49]
	Fatturi, 2020 [33]	85	107	63	76	43.2%	0.80 [0.37, 1.70]
	Madalena, 2020 [35]	60	79	7	9	8.6%	0.90 [0.17, 4.72]
Subtotal (95% CI)			269		182	100.0%	0.77 [0.46, 1.27]
Total events		209		150			
Heterogeneity: Chi² = 0.08, df = 2 (*P =* 0.96); I² = 0%; Test for overall effect: *Z* = 1.04 (*p* = 0.30)							
ff + Ff vs. FF	Barbosa, 2020 [31]	145	164	162	179	47.7%	0.80 [0.40, 1.60]
	Fatturi, 2020 [33]	182	204	119	132	41.4%	0.90 [0.44, 1.86]
	Madalena, 2020 [35]	119	138	17	19	10.9%	0.74 [0.16, 3.45]
Subtotal (95%CI)			506		330	100.0%	0.84 [0.52, 1.35]
Total events		446		298			
Heterogeneity: Chi² = 0.09, df = 2 (*P =* 0.96); I² = 0%; Test for overall effect: *Z* = 0.73 (*p* = 0.46)							
ff vs. FF + Ff	Barbosa, 2020 [31]	81	164	82	179	46.5%	1.15 [0.76, 1.76]
	Fatturi, 2020 [33]	97	204	56	132	41.8%	1.23 [0.79, 1.91]
	Madalena, 2020 [35]	59	138	10	19	11.8%	0.67 [0.26, 1.76]
Subtotal (95%CI)			506		330	100.0%	1.13 [0.84, 1.51]
Total events		237		148			
Heterogeneity: Chi² = 1.27, df = 2 (*P =* 0.53); I² = 0%; Test for overall effect: *Z* = 0.82 (*p* = 0.41)							

Abbreviation: CI, confidence interval.

**Table 10:** The results from meta-analysis of the association between *BglI (rs739837)* polymorphism and dental caries risk based on five genetic models.

Genetic Model	First Author, Publication Year	Case		Control		Weight	Odds Ratio
		Events	Total	Events	Total		M-H, Random, 95%CI
b vs. B	Barbosa, 2020 [31]	186	326	203	376	48.1%	1.13 [0.84, 1.53]
	Fatturi, 2020 [33]	199	426	112	242	45.2%	1.02 [0.74, 1.40]
	Madalena, 2020 [35]	120	198	16	24	6.7%	0.77 [0.31, 1.88]
Subtotal (95%CI)			950		642	100.0%	1.06 [0.86, 1.31]
Total events		505		331			
Heterogeneity: Chi² = 0.74, df = 2 (*P =* 0.69); I² = 0%; Test for overall effect: *Z* = 0.51 (*p* = 0.61)							
bb vs. BB	Barbosa, 2020 [31]	52	81	58	101	45.8%	1.33 [0.73, 2.43]
	Fatturi, 2020 [33]	49	112	27	63	48.1%	1.04 [0.56, 1.93]
	Madalena, 2020 [35]	34	47	5	6	6.1%	0.52 [0.06, 4.91]
Subtotal (95%CI)			240		170	100.0%	1.15 [0.75, 1.75]
Total events		135		90			
Heterogeneity: Chi² = 0.80, df = 2 (*P =* 0.67); I² = 0%; Test for overall effect: *Z* = 0.63 (*p* = 0.53)							
Bb vs. BB	Barbosa, 2020 [31]	82	111	87	130	40.7%	1.40 [0.80, 2.44]
	Fatturi, 2020 [33]	101	164	58	94	55.1%	1.00 [0.59, 1.68]
	Madalena, 2020 [35]	52	65	6	7	4.2%	0.67 [0.07, 6.03]
Subtotal (95% CI)			340		231	100.0%	1.15 [0.79, 1.67]
Total events		235		151			
Heterogeneity: Chi² = 1.00, df = 2 (*P =* 0.61); I² = 0%; Test for overall effect: *Z* = 0.71 (*p* = 0.48)							
bb + Bb vs. BB	Barbosa, 2020 [31]	134	163	145	188	40.9%	1.37 [0.81, 2.32]
	Fatturi, 2020 [33]	150	213	85	121	54.7%	1.01 [0.62, 1.64]
	Madalena, 2020 [35]	86	99	11	12	4.4%	0.60 [0.07, 5.05]
Subtotal (95%CI)			475		321	100.0%	1.14 [0.80, 1.62]
Total events		370		241			
Heterogeneity: Chi² = 1.06, df = 2 (*P =* 0.59); I² = 0%; Test for overall effect: *Z* = 0.73 (*p* = 0.46)							
bb vs. BB + Bb	Barbosa, 2020 [31]	52	163	58	188	53.1%	1.05 [0.67, 1.65]
	Fatturi, 2020 [33]	49	213	27	121	38.4%	1.04 [0.61, 1.77]
	Madalena, 2020 [35]	34	99	5	12	8.5%	0.73 [0.22, 2.48]
Subtotal (95%CI)			475		321	100.0%	1.02 [0.73, 1.42]
Total events		135		90			
Heterogeneity: Chi² = 0.30, df = 2 (*P =* 0.86); I² = 0%; Test for overall effect: *Z* = 0.11 (*p* = 0.91)							

Abbreviation: CI, confidence interval.

**Table 11:** Subgroup analyses based on ethnicity and genotyping method for *TaqI (rs731236)* polymorphism.

Variable (N)	t vs. T	tt vs. TT	Tt vs. TT	tt + Tt vs. TT	tt vs. TT + Tt
	OR (95%CI), *p*, I^2^	OR (95%CI), *p*, I^2^	OR (95%CI), *p*, I^2^	OR (95%CI), *p*, I^2^	OR (95%CI), *p*, I^2^
Ethnicity					
Caucasian (3)	1.02 (0.81, 1.29), 0.86, 2%	1.17 (0.72, 1.89), 0.53, 0%	0.96 (0.59, 1.56), 0.87, 36%	1.02 (0.64, 1.61), 0.94, 39%	1.20 (0.77, 1.87), 0.42, 0%
Asian (3)	0.85 (0.69, 1.05), 0.13, 0%	0.75 (0.05, 13.07), 0.87	0.72 (0.47, 1.10), 0.13, 0%	0.72 (0.47, 1.10), 0.13, 0%	0.59 (0.35, 1.00), 0.05
Genotyping method					
PCR (4)	0.99 (0.71, 1.37), 0.95, 27%	1.44 (0.72, 2.85), 0.30, 0%	0.91 (0.62, 1.33), 0.63, 14%	0.96 (0.64, 1.43), 0.83, 28%	1.23 (0.66, 2.29), 0.51, 0%
TaqMan (2)	0.89 (0.74, 1.08), 0.23, 0%	0.94 (0.48, 1.82), 0.85, 0%	0.71 (0.46, 1.09), 0.12, 0%	0.75 (0.49, 1.14), 0.17, 0%	0.81 (0.42, 1.58), 0.54, 62%

Abbreviations: OR, odds ratio; CI, confidence interval.
